# How “mood‐incongruent psychosis” was included under affective disorders in the DSM‐III

**DOI:** 10.1002/pcn5.70090

**Published:** 2025-03-31

**Authors:** Hiroyuki Takiue

**Affiliations:** ^1^ Department of Psychiatry Tokyo Metropolitan Matsuzawa Hospital Setagaya‐ku Tokyo Japan

**Keywords:** Karl Jaspers, ego‐psychology school, Feighner criteria, hierarchical principle inversion, paranoid reaction

## Abstract

The Diagnostic and Statistical Manual, Third Edition (DSM‐III), the prototype of the modern DSMs, differed from previous traditions in American psychiatry in that it was etiologically agnostic. It also represented a re‐importation of German psychiatry for the first time since Freud. An exception, however, was the hierarchical relationship between mood and psychosis, which was weighted in favor of mood in the United States. Specifically, mood‐incongruent psychosis was considered a symptom that could also occur in affective (mood) disorders. This was a decision that also differed from the Ninth Revision of the International Statistical Classification of Disease, which was published 3 years before the DSM‐III. It was not until the nineteenth century that a distinction was made between mood and psychosis. In Germany, the emphasis was on psychosis, whereas in the United States, under the strong influence of Adolf Meyer, the emphasis was on depression. Hallucinatory and delusional states, later summarized as Kurt Schneider's “first‐rank symptoms” (FRS), were also incorporated into the framework for mood disorders. Subsequently, the United States entered the heyday of psychoanalysis, while German descriptive psychopathology was difficult to accept. When the DSM‐III was drafted in the 1970s, the FRS were “cut out” from that descriptive psychopathology and introduced. However, since that time, the FRS, along with other mood‐incongruent psychoses, have lost their specificity for schizophrenia and organic psychosis.

## INTRODUCTION

The modern versions of the Diagnostic and Statistical Manual of Mental Disorders (DSM), beginning with the Third Edition (DSM‐III), are widely known to provide operational diagnostic criteria that do not focus on etiology, except for mental disorders with a clear physical or substance‐based basis or stress‐induced mental disorders such as posttraumatic stress disorder.^1^, ^2^, ^3^

However, they are also said to have another major feature. De Leon^4^ pointed out that affective symptoms (symptoms typical of mood disorders) rank above schizophrenia symptoms in the DSMs, which is inverted from the “hierarchical principle” (“*Hierarchieregeln*” in German) advocated by Karl Jaspers.^5^, ^6^ For more details, mood‐incongruent psychoses, including Kurt Schneider's “first‐rank symptoms” (FRS), are not pathognomonic for schizophrenia, but can be found in mood disorders.

Regarding a manic episode, the definition of “mood‐incongruent psychotic features” in the DSM‐III was:

Either (a) or (b):

(a) Delusions or hallucinations whose content does not involve themes of either inflated worth, power, knowledge, identity, or special relationship to a deity or famous person. Included are such symptoms as persecutory delusions, thought insertion, and delusions of being controlled, whose content has no apparent relationship to any of the themes noted above.

(b) Any of the following catatonic symptoms: stupor, mutism, negativism, posturing.^1^

Regarding a major depressive episode:

Delusions or hallucinations whose content does not involve themes of either personal inadequacy, guilt, disease, death, nihilism, or deserved punishment. Included here are such symptoms as persecutory delusions, thought insertion, thought broadcasting, and delusions of control, whose content has no apparent relationship to any of the themes noted above.^1^

The definition of “mood‐incongruent psychosis” in the present study is the same as that given above. Although the term was originally used only in affective (mood) disorders, for simplicity, it is also used in the context of schizophrenia in the present paper.

The main purpose of the adoption of “etiology‐agnostic” and atheoretical diagnostic criteria in the DSM‐III was to eliminate the psychoanalytic or psychodynamic theories that had dominated the United States up to that time.^7^ On the other hand, many of the diagnostic classifications in the DSM‐III were created in a hierarchical manner, which was clearly influenced by Schneider's diagnostic approach based on Jaspers' hierarchical principle.^8^ The fact that the DSM‐III did not follow Jaspers and Schneider on the relationship between mood disorders and schizophrenia could be considered an exceptional treatment. This issue has already been discussed by Kendler,^9^ but the present author could not identify any previous studies discussing its relationship with the history of American psychiatry.

The present paper reflects on what differences in thinking may result in the different handling of mood‐incongruent psychosis. In addition, this paper discusses the history of how mood‐incongruent psychosis was found to occur in mood disorders in the DSM‐III with reference to the history of American psychiatry in the first half of the twentieth century.

## HISTORY OF THE RELATIONSHIP BETWEEN MOOD AND PSYCHOSIS IN GERMAN DESCRIPTIVE PSYCHOPATHOLOGY

### Prehistory: Delusion had been separated from mood

The distinction between reason and emotion was not applied to the concept of delusion before the nineteenth century. Around 1900, psychiatrists began to distinguish delusion‐like ideas secondary to the affective state from primary delusions. For example, Wilhelm Griesinger once thought that delusions were logical secondary derivations of an affective background. He later changed his opinion: even if delusions and pathological mood coincide, that does not necessarily indicate a causal relationship between the two.^10^ On the other hand, in the late nineteenth century in the United States, some studies classified psychotic symptoms corresponding to the FRS as manic states.^11^

In the twentieth century, Jaspers distinguished between primary and secondary delusions, clearly stating that “delusion can only arise in the process of thinking and judging” on p. 95 of his literature.^6^, ^10^ His definition was accepted by the Heidelberg School, the most prominent psychiatry school in Germany (of which Jaspers and Schneider were members), and soon became the mainstream theory on delusions.^10^

### Categorization of mental disorders by cross‐sectional phenomenology or long‐term outcome results in different treatments for mood‐incongruent psychosis

According to German descriptive psychopathology, whether to allow mood‐incongruent psychosis to occur in mood disorders reflected a difference in position: classifying mental disorders on the basis of cross‐sectional phenomenology or long‐term outcomes. Schneider described the following:

The diversity met with in handling the differential diagnosis between schizophrenia and cyclothymia is really due to whether the investigator is laying decisive weight on the state of the patient or on the course of the complaint. Whoever, like us, emphasizes the condition will, if the psychosis shows any schizophrenic symptoms of first‐rank importance, hold firmly to such a diagnosis, although the psychosis clears up completely. But whoever diagnoses according to the course the illness takes will, if he cannot find some third term of his own, call such a psychosis a “phase of manic‐depressive insanity,” without taking into account its other perhaps more schizophrenic symptoms. In such atypical borderline cases an arbitrary diagnosis often has to be made, either of schizophrenia with an atypical course or of cyclothymia with atypical symptoms.^12^

When classified cross‐sectionally, any finding of mood‐incongruent psychosis, including the FRS, is diagnosed as schizophrenia, regardless of prognosis. On the other hand, classifying on the basis of prognosis is done to acknowledge that mood‐incongruent psychosis can also occur in mood disorders.

Regarding psychosis in cyclothymic depression (endogenous depression):

Delusions of sin, hypochondriacal delusions, and delusions of impoverishment are well recognized and prominent features. Their occurrence is not altogether by chance; the type of delusion is not a direct “symptom” of the psychosis but represents already existing primary human anxieties, unmasked by the depression but not positively produced by it. Anxieties about the soul, the body, and subsistence are basic human anxieties.^12^

Regarding schizophrenia:

Where the somatic nature of the psychosis is in doubt (authors' note: this is nearly equal to “endogenous psychosis”), there is an easy diagnosis nowadays of more or less typical cyclothymias, but everything else tends to get classed as schizophrenia. All the characteristics which will not fit into cyclothymic picture are gathered together under this one term.^12^

To note, Schneider's “cyclothymia” referred to both unipolar and bipolar mood disorders^12^ and differed from “cyclothymic disorder” in current psychiatry.

The assumption must be made that mood‐incongruent psychosis is a more severe finding than mood swings. Schneider's FRS were characterized as being markedly abnormal in their form of experience (i.e., they could be considered abnormal regardless of the content of their thoughts) and differentiated from cyclothymia.^12^ He carried on Jaspers' “hierarchical principle” in a virtual way.

By contrast, a typical example of a position that classifies from the course and prognosis is Emil Kraepelin.^13^ With the exception of his paper on the “patterns of mental disorder (Die Erscheinungsformen des Irreseins),”^14^ which he published in his later years in response to criticism from Hoche et al., he did not incorporate the idea of hierarchy.

### Jaspers proposed the hierarchical principle based on the concept of “explaining and understanding”

Jaspers' “hierarchical principle” clearly stated the diagnostic superiority of schizophrenia over bipolar disorder:

The position can be illustrated pictorially: the disease symptoms lie on different superimposed planes; the neurotic symptoms are on top (psychasthenic, hysteric), then come the manic‐depressive, then the process symptoms (schizophrenia), and finally the organic (psychic and somatic) symptoms.^6^

At the same time, he stated that this is only a hypothesis, as “where a schizophrenic process is present we duly suppose that we should hold it responsible for all the symptoms, but that is a presupposition.”^6^ Furthermore, he added that there could be an overlap between bipolar disorder and schizophrenia.^6^

This idea, like Schneider's, focused on cross‐sectional phenomenology. Schizophrenia was already ununderstandable on a “cross‐sectional” level (called “statistically ununderstandable (Statisch unverständlich)”) and considered “madness (Verrücktheit).” He distinguished schizophrenia from mood disorders, in which ununderstandable is only reached by tracing changes in symptoms over time. The original text is as follows:

(*d*) *Affective illnesses (Gemütskrankheiten) and mental illness (Geisteskrankheiten)* (natural and schizophrenic psychic life). The most profound distinction in psychic life seems to be that between what is meaningful and *allows empathy* and what in its particular way is *ununderstandable*, “mad” in the literal sense, schizophrenic psychic life (even though there may be no delusions). Pathological psychic life of the first kind we can comprehend vividly enough as an exaggeration or diminution of known phenomena and as an appearance of such phenomena without the usual causes or motives. Pathological psychic life of the second kind we cannot adequately comprehend in this way. Instead we find changes of the most general kind for which we have no empathy but which in some way we try to make comprehensible from an external point of view. Language has always differentiated *affective illness* from *madness* proper… Affective illnesses he indeed calls unmotivated but profound emotional disturbances for which, after their own fashion, one can have empathy, as for instance those of melancholia… 1. Looking at the *phenomenological elements* we find in morbid psychic life that there are those which can be seen under favourable circumstances, though they are difficult to discern at all clearly, and those which *can in principle never be seen by us*, which we always have to circumscribe negatively and indirectly by saying what they are not. Such elements which are in principle psychologically inaccessible we term “*statically ununderstandable*” or closed to empathy.^6^

Accordingly, mood‐incongruent psychosis, including the FRS, is incorporated into schizophrenia.

The concept of “explaining and understanding” proposed by Jaspers became the foundation of German descriptive psychopathology when approaching the patient. For Jaspers, the basic attitude to face the patient and determine whether the mental state actually experienced is understandable is as follows:

Phenomenology sets out on a number of tasks: it gives a concrete description of the psychic states which patients actually experience and presents them for observation. It reviews the inter‐relations of these, delineates them as sharply as possible, differentiates them and creates a suitable terminology.^6^

In addition, the static understanding “denotes the presentation to oneself of psychic states, the objectifying to oneself of psychic qualities.”^6^

On the other hand, in the first edition published in 1913,^5^ Jaspers also discussed the relationship between mood disorders and schizophrenia in terms of a long‐term prognosis, similar to Kraepelin. He positioned schizophrenia as “disease processes” that begin at a certain time, remain incurable, lead to a permanent transformation of at least one side of the personality, and usually present the psychological characteristic of schizophrenic psychic life, as well as a more serious illness than bipolar disorder (at the time, Jaspers defined bipolar disorder as “degenerative psychosis (degenerative Irresein)”).^5^ This section was removed in later editions (and thus has never been translated into English). Schneider's earlier view of diagnosing on the basis of cross sections^15^, ^16^ might have influenced Jaspers.^17^

### Jaspers' hierarchical principle in subsequent Anglophone psychiatry

As already discussed,^18^, ^19^ Anglophone psychiatry rarely mentioned Jaspers. It seems that the same was true for his hierarchical principle. First, there was no mention of the hierarchical principle at the time of the Mayer‐Gross et al. textbook,^20^ which is said to have introduced German descriptive psychopathology to Anglo‐Saxon psychiatry.^4^ Moreover, in the chapter on the hierarchical principle in the book *One century of Karl Jaspers' general psychopathology*, ^21^ Jaspers’ hierarchical principle itself is explained, but its subsequent treatment in the English‐speaking world is not. Even in the philosophical discussion of the psychiatric classification,^22^ Jaspers was not mentioned except for the discussion about personality disorders. Despite the DSM‐5's residual hierarchy between organic and other mental disorders,^8^ except for Ghaemi ^23^ and so on, the question of what that hierarchy is seems to have been left open.

## THE PROCESS OF CREATING “MOOD‐INCONGRUENT PSYCHOSIS” OF AFFECTIVE DISORDERS IN THE DSM‐III, TRACED THROUGH THE HISTORY OF PSYCHIATRY IN THE UNITED STATES

### The process by which mood‐incongruent psychosis was determined to occur in affective disorders in the DSM‐III

The DSM‐III, unlike the DSM‐II, is often presented as rejecting psychoanalytic theory, being etiologically independent, and being strongly influenced by “neo‐Kraepelinians” such as Eli Robins and Samuel Guze through their Feighner criteria.^4^, ^24^ In addition, according to de Leon,^4^ in the DSM‐III, Jaspers' hierarchy of mood disorders and schizophrenia was inverted based on the results of Pope and Lipinski.^25^

Moreover, the Ninth Revision of the International Statistical Classification of Disease (ICD‐9), published 3 years before the DSM‐III, stated that only mood‐congruent psychosis occurs in affective disorders: “delusions, perplexity, disturbed attitude to self, disorder of perception and behaviour; these are all in keeping with the patient's prevailing mood [as are hallucinations when they occur].”^26^ Thus, the determination that mood‐incongruent psychosis occurs in affective (mood) disorders might be original to the United States.

How, in fact, was mood‐incongruent psychosis determined in the DSM‐III to also occur in affective disorders? Pope and Lipinski concluded that there were no pathognomonic symptoms for schizophrenia and that classical “schizophrenic” symptoms, including the FRS, were also reported in 20%–50% of well‐validated cases of manic‐depressive illness. Moreover, “in patients with some affective symptoms and, particularly, good functioning between psychotic episodes, presence or absence of ‘schizophrenic’ symptoms has no demonstrated effect on response to lithium carbonate.”^25^

Spitzer (the lead editor of the DSM‐III) et al.^27^ concluded that “given our current knowledge (ignorance?), some of Schneider's first rank symptoms are useful in the diagnostic criteria, provided that both an Organic Mental Disorder and an affective syndrome have been ruled out.” The debate over where to draw the line between schizophrenia and affective disorders, especially schizoaffective disorder (SD), was extremely contentious (some authorities thought that SD should be classified as an affective disorder).^28^ For SD, Spitzer et al.^27^ had to make “a compromise between two extremes: those who consider it a subtype of Schizophrenia and those who consider it a form of Affective Disorder (Pope and Lipinski 1978).”

In the process of reaching their conclusion, Spitzer et al.^27^ mainly cited the results of Carpenter et al.^29^, ^30^, ^31^ (Carpenter was a member of the DSM‐III's Multiaxial Diagnosis Advisory Committee^28^). The diagnostic criteria used in these studies were the DSM‐II or ICD‐8. At least in the classification of schizophrenia and affective disorders, the DSM‐II and ICD‐8 were nearly identical.^32^, ^33^ Moreover, “the ICD‐8 was only a list of approved diagnostic terms. No attempt was made to define the terms in the ICD‐8.”^34^

Thus, rather than being an inversion of the hierarchical principle via Pope and Lipinski,^25^ the decision regarding mood‐incongruent psychosis in the DSM‐III was carried over from studies using the DSM‐II. The following sections discuss how the DSM‐II was created and used.

### Adolf Meyer emphasized depression and incorporated the FRS into his “affective reaction”

Adolf Meyer had a strong influence on American psychiatry in the first half of the twentieth century. Although his discussion is extremely difficult to understand, and it is not always clear what interpretation is correct,^35^ the points related to this paper are examined as follows.

Meyer emphasized depression, the influence of which remains strong in modern American psychiatry.^23^ He wrote:

“Depressions” would be a better designation for the large group of types, each of which would be distinguished according to etiology, symptom complex, course of illness, and results; according to the intrinsic nature of the depression. There were pronounced types, such as manic‐depressive depressions, anxiety psychoses, depressive deliriums, depressive hallucinosis, depressive episodes of dementia praecox, and symptomatic depressions, as well as non‐differentiated types.^36^

Whether schizophrenia or delirium, he considered any depression to be “Depression”.

Meyer's mother was experiencing delusional depression,^35^ and this family history might have been at the root of his decision. This fact, along with his visit with Kraepelin in Heidelberg, led Meyer to turn from neuropathology to clinical psychiatry.^35^ Meyer also wrote:

The mere fact that depression, of whatever origin, is apt to be dangerous as a foundation for suicide and tends to shut in the patient in self‐absorption so as to exclude the corrective influences of the environment and so as to allow a cropping‐out of uncorrected ideas and developments which may take a progressive character, of the nature of a vicious circle, especially where for some reason constitutional safeguards are lacking.^36^

Meyer initially relied on Kraepelin's thinking; however, he gradually changed his idea:

He persistently swam against the rising tide of Kraepelin's descriptive psychiatry and tried to counter demands for standardized terminology. In Outlines of Examinations, he warned readers that “without some qualifications, more than one half of the cases are not sufficiently clean‐cut to represent a type.”^35^

He became more concerned with patterns of development and reactions than with diagnoses.

During this process, Meyer's concept of “reaction” was generated. In 1908, Meyer described three general reaction types: organic, affective, and substitute. Organic reactions generally corresponded to organic psychosis, whereas affective reactions included “paranoid reactions” as well as depression:

A paranoid reaction might comprise various degrees of disordered thinking, from unwarranted suspiciousness to systematized delusions. A sufferer might suggest that the conversation of strangers or the flickering of street lamps referred to his or her own person. Driven by pathological emotional cues to hide, commit suicide, or attack, patients exhibiting an affective reaction engaged in unaccountable, unpredictable, and often reckless behavior.^35^

In other words, at least one of the FRS (the above example would represent a “delusional perception”) was categorized as an affective reaction. On the other hand, schizophrenia was incorporated into the substitutive reaction, along with hysteria and so on,^35^ with his understanding that it is a “habit disorder.”^37^ Schizophrenia in German psychiatry was divided into affective and substitutive reactions. Perhaps he used pre‐nineteenth‐century theories, before the separation of emotions and delusions, as a support.

Meyer's influence also extended to the first edition of the DSM, published in 1952 (2 years after his death).^38^ The DSM “was a catalogue of Meyerian reaction‐types.”^35^ At the same time, “the prominence of psychodynamic and psychoanalytic concepts in DSM‐I”^39^ was noted. Moreover, the DSM‐II, published in 1968, was similar to the DSM‐I, although the word “reaction” was lost.^40^ In fact, the DSM‐I had a classification of “Paranoid Reactions,” which corresponded to the “Paranoid states” in the DSM‐II (however, Paranoid states were distinguished from not only schizophrenia, but also affective psychoses in the DSM‐II).^32^

### The “explaining and understanding” concept and nosology were marginalized in American psychiatry under the dominance of the psychodynamic paradigm

Meyer's viewpoint that normality and abnormality were on a continuum and his emphasizing “reaction” were high affinity for dynamic psychiatry.^35^, ^41^ Given this background, psychoanalysis emerged in American psychiatry following events such as lectures in the United States by Sigmund Freud in 1909.

As psychoanalysis developed in the United States, it split into several styles. The mainstream ego‐psychology school incorporated the ideas of Freud.^41^, ^42^ Heinz Hartmann, who was one of the main figures of the ego‐psychology school^42^ (and known as “the American prime minister of analysis”^43^), dismissed the significance of empathy because “the nature of empathic experience is still poorly understood, and its value in knowing other minds is still quite uncertain,”^41^, ^44^ while mentioning that Jaspers' “understanding” involved empathy.^44^ Under their supervision, “empathetic understanding” was introduced as an example demonstrated by “some trainees who have trouble working effectively.”^45^

Although empathy and understanding are not equivalent, it is impossible to understand a patient in the sense of Jaspers without empathy. If one does not seek to empathize with the patient, then the question of whether one can understand the patient is eliminated from the beginning.

By the mid‐1950s, nearly every department chairman of psychiatry in the United States was an advocate of psychoanalysis^18^ (the psychiatric nosology of America had already fallen into chaos by the late 1940s^43^): “In the heyday of psychoanalysis many psychiatrists were simply indifferent to diagnosis, and believed that ascertaining the presumed psychodynamic cause was more important than classifying the presenting symptoms.”^43^

Under such an environment, some psychoanalysts might even have introduced Jaspers' “explaining and understanding”; however, neither the concept regarding how much of the patient is understandable and how much is not, nor the approach of considering differential diagnoses based on such a concept was widely accepted anywhere.

### The interpretation of the early DSMs, which were not thoroughly operationalized, was left to the discretion of the practitioner, which could have led to the persistence of traditional American psychiatric practice

The nomenclature of the DSM‐II as well as the DSM‐I mirrored the psychoanalysts' predominance of the drafting committee.^43^ As a result:

The DSM‐I and DSM‐II made little effort to provide elaborate classification schemes, because overt symptoms did not reveal disease entities but disguised underlying conflicts that could not be expressed directly.^38^

Descriptions of all disorders were brief and usually lacking in operational criteria for their application. Psychiatrists seeking guidance in differentiating, for example, schizophrenia from mania would find little to help them.^43^

With the DSM‐II's lack of formal criteria for determining diagnostic boundaries, clinicians were forced to rely on global descriptions of disorders that often entailed subjective assumptions about a disorder's cause.^38^

Diagnosis using the DSM‐II (and ICD‐8) left space for unstated, inherited practices and interpretations by each psychiatrist. Given Meyer's influence, using the DSM‐II, anyone with depression would have been classified as having an affective disorder even if they had the FRS. If this patient presented with psychomotor excitation based on hallucinations and delusions, that patient would have been considered manic and thus diagnosed with bipolar disorder. Without such a presumption, it is very difficult for the author, a Japanese psychiatrist, to explain Pope et al.'s finding that as many as 20%–50% of bipolar patients have classic “schizophrenic” symptoms, including the FRS.^25^ Kraepelin also stated that:

It is common to find cases of undeniable schizophrenia which show transitory or at times longer‐lasting manic‐depressive symptoms, and it may be quite impossible to distinguish them from the circular forms of insanity. Schizophrenic symptoms are found to develop in the course of manic‐depressive insanity much less frequently.^14^

Certainly, the text of the DSM‐II did not imply that mood symptoms were diagnostically more relevant than symptoms in thought or perception. As Kendler mentioned,^9^ the notation “When illusions, hallucinations, and delusions (usually of guilt or of hypochondriacal or paranoid ideas) occur, they are attributable to the dominant mood disorder” was regarded as diagnostic criteria for manic‐depressive illness, depressed type.^32^ However, considering that Meyer included at least some of the FRS in the affective reactions, there is still room for the FRS to be considered “attributable to the dominant mood disorder” (i.e., mood‐congruent psychosis) and included as affective (mood) disorders (if this notation had been interpreted as defined by the German descriptive psychopathology, it would not have resulted in studies showing that the FRS were present in a large number of patients with affective (mood) disorders). In addition, there was no such notation in the criteria of manic‐depressive illness, manic type.^32^ Therefore, it was possible to consider mood‐incongruent psychosis as a manic state. Actually, Taylor et al. made such an argument:

We suggest the prior overdiagnosis of schizophrenia resulted from failure to adequately weight cardinal phenomena of mania when they occurred in the presence of phenomena thought to be specific for the diagnosis of schizophrenia.^46^

Because the DSM‐II was rarely used in clinical practice,^47^ there was probably no debate over its usage in daily clinical settings.

On the other hand, Meyer included most patients with schizophrenia in the same “reaction” group as those with hysteria. Both this and the indifference to nosology under the influence of psychoanalysis would have led to a distinct preponderance of schizophrenia diagnoses in the United States. For example, 69% of American psychiatrists diagnosed a young man who had hysterical paralysis of one arm and a history of mood fluctuations associated with alcohol abuse as having “schizophrenia.”^43^ Finally, “according to US psychiatrists trained in psychoanalytic theory before the 1980s, almost any patient who had psychosis or severe mental illness had schizophrenia.”^18^

A US–UK study^48^ identified an overdiagnosis of schizophrenia and an underdiagnosis of bipolar disorder in the United States. Although Taylor et al.^46^ argued that the overdiagnosis of schizophrenia is partly due to the belief that mood‐incongruent psychosis does not occur in affective (mood) disorders, this belief was shared in the ICD‐9.

By contrast, it remains unclear what caused the underdiagnosis of bipolar disorder. At least in light of the American originality of the decision to include mood‐incongruent psychosis as an affective (mood) disorder, it would not be possible to determine that the underdiagnosis was related to mood‐incongruent psychosis. Perhaps a manic state with mood‐congruent psychosis could have been misdiagnosed as schizophrenia in the US–UK study.^48^

### Feighner criteria indicated that a diagnosis of schizophrenia could not be made without ruling out affective episodes

As previously mentioned, the Feighner criteria^24^ were used as a reference in the development of the DSM‐III. For the diagnosis of schizophrenia, both of the following criteria were necessary: (1) a chronic illness with at least 6 months of symptoms prior to the index evaluation without return to the premorbid level of psychosocial adjustment, and (2) absence of a period of depressive or manic symptoms sufficient to qualify for affective or probable affective disorder.

This exclusion seemed to be due to Robins:

The point of departure in Dr. Robins' thinking was that schizophrenia was a deteriorating disorder. Dr. Robins, a student of Wing, Schneider, Leonhard, Fish, and others, became convinced that the symptoms they addressed had no prognostic value and were often seen in affective disorders with psychosis… Dr. Robins thought schizophrenia could be diagnosed only in patients who had had symptoms for a long time and were presenting manifestations of social and occupational deterioration. He insisted that the course of the illness was much more important than the character of the delusions and hallucinations.^49^

Robins' career seems to have been exclusively developed in the United States.^28^ The context of his ideas is not clear, but it is possible that he reflected the thinking in the country at the time regarding the distinction between schizophrenia and mood disorders, except for Kraepelin's.

## DISCUSSION

It was through Wing's evidence using the Present State Examination (PSE, based on Schneider and Mayer‐Gross' work) that Spitzer incorporated the FRS into the DSM‐III.^50^ However, the FRS, which were supposed to be “markedly different from cyclothymias (mood disorders),” were accepted from the beginning in American psychiatry as “also found in mood disorders.” Without comprehending the underlying background of the descriptive psychopathology represented by Jaspers' “explaining and understanding” (which may have been influenced by the ego‐psychology school), it appears that only the FRS were cut out and imported to the United States.

Other misconceptions can be cited. Because Schneider emphasized cross‐sectional phenomenology in his classification, it is not a question of whether the FRS are predictive of prognoses.^12^ Classification based on long‐term prognoses changes the entire relationship between mood disorders and schizophrenia. The argument that the FRS are not useful for diagnosis because they cannot be used to predict prognosis^51^ is, in fact, merely a reaffirmation of the old fact that the state picture does not predict prognosis: “symptoms of first‐rank importance cannot be used for purposes of prognosis.”^12^

It was impossible to give equal weight to both the phenomenology and longitudinal prognosis in the diagnosis. In addition, it is not possible to determine that the idea that emphasizes one of them is right and the other is wrong (the same is true for the question of whether mood‐incongruent psychosis or mood swings should be considered a more severe finding). Moreover, prognosis‐based classification does not rule out the possibility that some patients with schizophrenia have a good prognosis.

While nosology became more chaotic, there appears to have been a tradition in the pre‐DSM‐III America of including mood‐incongruent psychosis in affective (mood) disorders as a result of Meyer's influence (to the best of the author's knowledge, no one other than Meyer has had a major influence on this “tradition”). Otherwise, the Feighner criteria for diagnosing schizophrenia would not have included “exclusion of affective disorders,”^24^ nor would several experts have expressed a strong belief that schizoaffective disorder belongs to affective disorders during the DSM‐III development process.^28^ The fact that it was carried over into the DSM‐III meant, perhaps unintentionally, that the operative diagnostic criteria, which emphasized cross‐sectional aspects, were mixed with a perspective that emphasized long‐term prognoses. It might have appeared as if it were a “inversion” of the hierarchical principle.

Furthermore, according to Spitzer et al.,^27^ the address of mood‐incongruent psychosis in the DSM‐III was tentative. This address is just a declaration. The evidence standing for that manual does not rule out the possibility that there is no mood‐incongruent psychosis in mood disorders because the evidence cannot prove the correctness of the manual on which it stands.

The continuation of the current situation could have a negative impact on the examination of the biological relationship between schizophrenia and mood disorders. Although consensus has been reached that these share a common genetic basis, Jaspers introduced Luxenburger's study (Luxenburger “undertook the first large‐scale survey of all twins born in a given area” as a psychiatrist^43^), showing that it was unlikely that both schizophrenia and bipolar disorder were found in the same family.^6^ Moreover, one reason why atypical antipsychotics are effective for mood disorders may be the acceptance that mood‐incongruent psychosis can occur in mood disorders. One recent study suggested that bipolar disorder with mood‐incongruent psychosis has a worse prognosis (more akin to schizophrenia, if based on Kraepelin's definition) than bipolar disorder without it.^52^

On the other hand, in the process of creating the DSM‐5, the adoption of a dimension‐based diagnostic classification rather than a categorical format was considered.^53^ If future DSMs and ICDs move to a dimensional format, as Kumazaki pointed out,^10^ the hierarchical relationship between mood and psychotic symptoms may disappear.

The question of whether mood swings or psychosis should be considered a more serious condition is a philosophical conundrum. Ghaemi may have correctly observed the issue surrounding the middle ground between schizophrenia and bipolar disorder: “clearly, the argument is partially a disagreement on underlying philosophy of science.”^23^ In conclusion, even with evidence‐based medicine, one would not be able to escape the question of what theory or perspective one relies on to distinguish between mood disorders and schizophrenia.

## AUTHOR CONTRIBUTION

**Hiroyuki Takiue**: Conceptualization; investigation; methodology; writing—original draft; writing—review and editing.

## CONFLICT OF INTEREST STATEMENT

The author declare no conflicts of interest.

## ETHICS APPROVAL STATEMENT

N/A.

## PATIENT CONSENT STATEMENT

N/A.

## CLINICAL TRIAL REGISTRATION

N/A.

## Data Availability

N/A.

## References

[1] American Psychiatric Association . Diagnostic and statistical manual of mental disorders. 3rd ed. Washington, DC: American Psychiatric Association; 1980.

[2] American Psychiatric Association . Diagnostic and statistical manual of mental disorders. 4th ed. Washington, DC: American Psychiatric Association; 1994.

[3] American Psychiatric Association . Diagnostic and statistical manual of mental disorders. 5th ed. Text Revision. Washington, DC: American Psychiatric Association; 2022.

[4] de Leon J . Reflections on US psychiatry: how the baton was passed from european psychiatry and the contributions of US psychiatry. J Nerv Ment Dis. 2021;209(6):403–408. 34037550 10.1097/NMD.0000000000001324

[5] Jaspers K . Allgemeine Psychopathologie. Ein Leitfaden für Studierende, Ärzte und Psychologen. Berlin: Springer; 1913.

[6] Jaspers K . General psychopathology. (Trans. from German by Hoenig J and Hamilton MH with a new foreword by McHugh PR). Baltimore and London: Johns Hopkins University Press; 1997.

[7] Ohmae S . [The difference between depression and melancholia: two distinct conditions that were combined into a single category in DSM‐III]. Seishin shinkeigaku Zasshi. 2012;114(8):886–905 (in Japanese). 23012851

[8] Fukushima T , Harima H . Seishinbyouri no kaisougensoku to sousatekishindan. [The hierarchy principles of psychopathology and operationalized diagnoses]. Rinsho Seishin Igaku. 2014;43(2):195–199 (in Japanese).

[9] Kendler KS . Mood‐incongruent psychotic affective illness. A historical and empirical review. Arch Gen Psychiatry. 1991;48(4):362–369. 2009036 10.1001/archpsyc.1991.01810280078012

[10] Kumazaki T . What is a “mood‐congruent” delusion? History and conceptual problems. Hist Psychiatry. 2011;22(87 Pt 3):315–331. 22043664 10.1177/0957154X10390438

[11] Kendler KS , Mishara A . The prehistory of Schneider's First‐Rank Symptoms: texts from 1810 to 1932. Schizophr Bull. 2019;45(5):971–990. 31206162 10.1093/schbul/sbz047 PMC6737481

[12] Schneider K . Clinical psychopathology. (Trans by Hamilton MW). 5th ed. Oxford, England: Grune & Stratton; 1959.

[13] Kraepelin E . Psychiatrie: ein Lehrbuch für Studierende und Ärzte. III. Leipzig: Verlag von Johann Ambrosius Barth; 1913.

[14] Kraepelin E. Die erscheinungsformen des Irreseins (translated by H Marshall as: Patterns of mental disorder. In: Themes and Variations in European Psychiatry: An anthology. Eds S.R. Hirsch & M. Shepherd . Wright, Bristol, pp7‐30, l974). Z Gesamte Neurol Psychiatr. 1920;62(1):1–29.

[15] Schneider K . Psychischer Befund und Psychiatrische Diagnose. Leipzig: Thieme; 1939.

[16] Ohmae S . Shiken ni denai Schneider: “rinshoseishinnbyourigaku” shuui. [Schneider not on the exam: “Clinical Psychopathology” gleanings]. Seishin Igakushi Kenkyu. 2024;28(1):5–20 (in Japanese).

[17] Jäger M . Konzepte der Psychopathologie Von Karl Jaspers zu den Ansätzen des 21. Jahrhunderts. Stuttgart: W. Kohlhammer GmbH; 2015.

[18] de Leon J . One hundred years of limited impact of Jaspers’ General Psychopathology on US psychiatry. J Nerv Ment Dis. 2014;202(2):79–87. 24469517 10.1097/NMD.0000000000000092

[19] Tamada Y , Ohmae S . Eigo de yomu Jaspers, eigoken seishinigaku ni okeru “seishinbyorigaku souron” no ichiduke. [Karl Jaspers’ “Allgemeine Psychopathologie” in Anglophone psychiatry]. Rinsho Seishin Igaku. 2014;43(2):207–215 (in Japanese).

[20] Mayer‐Gross W , Slater E , Roth M . Clinical psychiatry. London: Cassell and Company Ltd; 1955.

[21] Sass H , Volpe U . Karl Jaspers' hierarchical principle and current psychiatric classification. In: Stanghellini G , Fuchs S editors. One century of Karl Jaspers' general psychopathology. Oxford: Oxford University Press; 2013. p. 185–207.

[22] Zachar P . A metaphysics of psyhopathology. Cambridge, MA: MIT Press; 2014.

[23] Ghaemi SN . The concepts of psychiatry: a pluralistic approach to the mind and mental illnes. Baltimore: Johns Hopkins University Press; 2007.

[24] Feighner, JP , Robins, E , Guze, SB et al. Diagnostic criteria for use in psychiatric research. Arch Gen Psychiatry. 1972;26(1):57–63. 5009428 10.1001/archpsyc.1972.01750190059011

[25] Pope Jr. HGLipinski Jr. JF . Diagnosis in schizophrenia and manic‐depressive illness: a reassessment of the specificity of “schizophrenic” symptoms in the light of current research. Arch Gen Psychiatry. 1978;35(7):811–828. 354552 10.1001/archpsyc.1978.01770310017001

[26] World Health Organization . International Conference for the Revision of the International Classification of Diseases (9th: 1975: Geneva). Manual of the international statistical classification of diseases, injuries, and causes of death: based on the recommendations of the Ninth Revision Conference, 1975, and adopted by the Twenty‐ninth World Health Assembly, Volume 1. Geneva: World Health Organization; 1977.

[27] Spitzer RL , Andreasen NC , Endicott J . Schizophrenia and other psychotic disorders in DSM‐III. Schizophr Bull. 1978;4(4):489–511. 734363 10.1093/schbul/4.4.489

[28] Decker H . The making of DSM‐III® A diagnostic manual's conquest of American psychiatry. New York: Oxford University Press; 2013.

[29] Carpenter Jr. WT , Strauss JS , Bartko JJ . Flexible system for the diagnosis of schizophrenia: report from the WHO International Pilot Study of Schizophrenia. Science. 1973;182(4118):1275–1278. 4752222 10.1126/science.182.4118.1275

[30] Carpenter Jr. WT . Are there pathognomonic symptoms in schizophrenia? An empiric investigation of Schneider's first‐rank symptoms. Arch Gen Psychiatry. 1973;28(6):847–852. 4707991 10.1001/archpsyc.1973.01750360069010

[31] Carpenter Jr. WT , Strauss JS . Cross‐cultural evaluation of Schneider's first‐rank symptoms of schizophrenia: a report from the International Pilot Study of Schizophrenia. Am J Psychiatry. 1974;131(6):682–687. 4827800 10.1176/ajp.131.6.682

[32] The Committee on Nomenclature and Statistics of the American Psychiatric Association . Diagnostic and statistical manual of mental disorders. 2nd ed. Washington, DC: American Psychiatric Association; 1968.

[33] World Health Organization . International Conference for the Eighth Revision of the International Classification of Diseases. Manual of the international statistical classification of diseases, injuries, and causes of death: based on the recommendations of the Eighth Revision Conference, 1965, and adopted by the Nineteenth World Health Assembly, Volume 1. Geneva: World Health Organization; 1967.

[34] Blashfield RK , Keeley JW , Flanagan EH , Miles SR . The cycle of classification: DSM‐I through DSM‐5. Annu Rev Clin Psychol. 2014;10:25–51. 24679178 10.1146/annurev-clinpsy-032813-153639

[35] Lamb SD . Pathologist of the mind: Adolf Meyer and the origins of American psychiatry. Baltimore, MD, US: Johns Hopkins University Press; 2014.

[36] Meyer A , Lief A . The commonsense psychiatry of Dr. Adolf Meyer: fifty‐two selected papers, with biographical narrative. New York, Toronto, London: Kessinger Publishing; 1948.

[37] Lamb S . Social skills: Adolf Meyer's revision of clinical skill for the new psychiatry of the twentieth century. Med Hist. 2015;59(3):443–464. 26090738 10.1017/mdh.2015.29 PMC4597240

[38] Mayes R , Horwitz AV . DSM‐III and the revolution in the classification of mental illness. J Hist Behav Sci. 2005;41(3):249–267. 15981242 10.1002/jhbs.20103

[39] Grob GN . Origins of DSM‐I: a study in appearance and reality. Am J Psychiatry. 1991;148(4):421–431. 2006685 10.1176/ajp.148.4.421

[40] American Psychiatric Association [Internet] . DSM history. Washington, DC: American Psychiatric Association. Available from: https://www.psychiatry.org/psychiatrists/practice/dsm/about-dsm/history-of-the-dsm

[41] Kitamura T . Kyokan to Seishinnbunnseki [Empathy and psychoanalysis]. Tokyo: Misuzu Shobo; 2021 (in Japanese).

[42] Richards AD . Psychoanalytic discourse at the turn of our century: a plea for a measure of humility. J Am Psychoanal Assoc. 2003;51(Suppl):73–89, discussion 125. 14761002

[43] Shorter E . A history of psychiatry: from the era of the asylum to the age of Prozac. Oxford, England: John Wiley & Sons; 1997.

[44] Hartmann H . Understanding and Explanation. Essays on ego psychology; selected problems in psychoanalytic theory. New York: International Universities Press; 1964. p. 369–403.

[45] Berlin IN . Some implications of ego psychology for the supervisory process. Am J Psychother. 1960;14:536–544. 13799544 10.1176/appi.psychotherapy.1960.14.3.536

[46] Taylor MA . The phenomenology of mania: a new look at some old patients. Arch Gen Psychiatry. 1973;29(4):520–522. 4748312 10.1001/archpsyc.1973.04200040066011

[47] Lieberman JA , Ogas O . Shrinks: the untold story of psychiatry. New York: Little, Brown Spark; 2015.

[48] Gurland BJ , Fleiss JL , Sharpe L , Roberts P , Cooper JE , Kendell RE . Cross‐national study of diagnosis of mental disorders: hospital diagnoses and hospital patients in New York and London. Compr Psychiatry. 1970;11(1):18–25. 5411210 10.1016/0010-440x(70)90200-2

[49] Kendler KS , Muñoz RA , Murphy G . The development of the Feighner criteria: a historical perspective. Am J Psychiatry. 2010;167(2):134–142. 20008944 10.1176/appi.ajp.2009.09081155

[50] Kendler KS . An historical framework for psychiatric nosology. Psychol Med. 2009;39(12):1935–1941. 19368761 10.1017/S0033291709005753 PMC2783473

[51] Carpenter WT , Strauss JS . Ideology and scientific progress: first‐rank symptoms. Schizophr Bull. 2019;45(5):950–951. 31508803 10.1093/schbul/sbz072 PMC6737472

[52] Shi HM , Jiang DG . A two‐year follow‐up study on the first manic episode due to mood‐incongruent psychosis. Asian J Psychiatr. 2022;73:103118. 35468481 10.1016/j.ajp.2022.103118

[53] Narrow WE , Kuhl EA . Dimensional approaches to psychiatric diagnosis in DSM‐5. J Ment Health Policy Econ. 2011;14(4):197–200. 22345361

